# ‘To Market to Market…’ and Risk for Global Disease

**DOI:** 10.3201/eid1308.000000

**Published:** 2007-08

**Authors:** Polyxeni Potter

**Affiliations:** * Centers for Disease Control and Prevention, Atlanta, Georgia, USA

**Keywords:** Constant Troyon, Barbizon school, landscape painting, animal painting, literature and medicine, cattle painting, On the Way to the Market, art and science connection, about the cover

**Figure:**
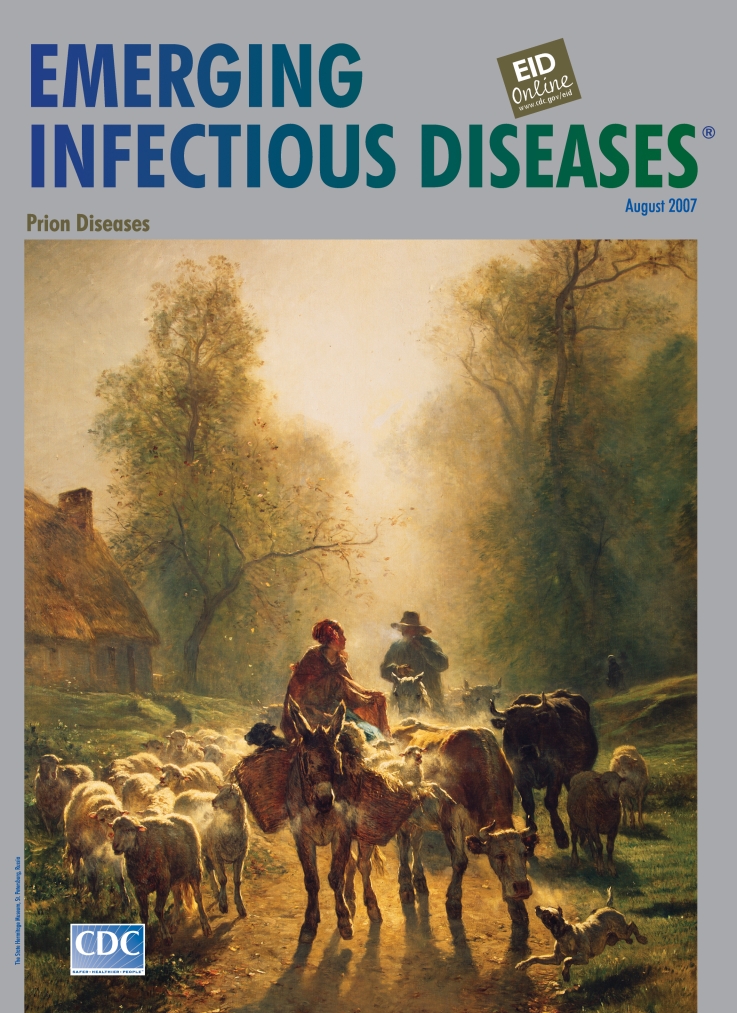
**Constant Troyon (1810–1865). On the Way to the Market (1859).** Oil on canvas (260.5 cm × 211 cm). State Hermitage Museum, St. Petersburg, Russia (to be verified when we have the image)

“…[T]he sound of water escaping from mill-dams…old rotten planks, slimy posts, and brick work, I love such things…. As long as I do paint, I shall never cease to paint such places. They have always been my delight,” wrote John Constable (1776–1837), English landscape painter and source of inspiration for Constant Troyon and others, who looked for subjects not in the classical or academic tradition but in natural surroundings (*1*).

Bucolic farms, rivers, trees, animals, and the common people populated 19th-century French painting as fields, streams, and rural life seemed palatable alternatives to the ravages of industrial development. Artists, among them Charles-François Daubigny, Théodore Rousseau, Jules Dupré, Narciso Diaz del la Peña, rejecting the urban scene, migrated to the countryside south of Paris, near Fontainebleau Forest, to live and work in the small village of Barbizon. There, eschewing modernity and its unwelcome transformations, they sought their own style and in the process laid the foundation for realism and, later, impressionism (*2*).

The Barbizon school, as these artists became known, took the studio outdoors, in *plein air*, where the landscape ceased to be just the backdrop of classical or historical scenes and became a subject in its own right. And, recalling 17th-century Dutch traditions, it contained animals and peasant farmers engaged in everyday activities.

Among the first Barbizon artists to become successful, Troyon became known as one of the best animal painters of his time. A native of Sèrves, he learned painting at the porcelain manufactory, where his father and grandfather were painters. Porcelain painting served him well, not for its exacting technique and judicious use of color alone but as back-up whenever he needed support to travel with other landscapists (Louis Cabat, Camille Roqueplan) and to paint the countryside around Sèrves and farming landscapes of Brittany and Normandy.

In the 1830s, Troyon started exhibiting in the Salon, where his landscapes attracted much attention among experts and the public. He was able to travel to Holland and see the masterpieces of the Dutch Golden Age. Inspired by works of Aelbert Cuyp, Esaias van de Velde, and Paulus Potter, he became more and more interested in painting animals: sheep flocks on country roads, oxen in plowing scenes. In his paintings, often large and imposing, landscapes were identified by the animals they sustained: Normandy by dairy cows grazing the lowlands, Fontainebleau by hunting dogs, recalling royal hunting parties at a nearby chateau (*3*). These tranquil landscapes, painted in a casual unaffected style, gained him popularity abroad.

Some of Troyon’s later works, among them On the Way to the Market, on this month’s cover, were recognized masterpieces. He was decorated with the Legion of Honor and counted Napoleon III as his patron. His last exhibit in the Salon was in 1859. He became ill with the paralytic symptoms of venereal infection. The disease progressed to fits of madness for which he was confined to an asylum. He died soon afterwards (*4*).

The rise of landscape painting in the midst of industrial development in 19th-century France reflected general angst about the intrusion of machines and disruptions of constant change. For the Barbizon painters, the landscape was an opportunity to be in nature, probe its mysteries, “…lie on fern or withered heath,” find “better worlds” (*5*).

So it was for Troyon, who viewed nature as a sanctuary, where animals and humans could live harmoniously. On the Way to the Market is a wistful image of country life at its peaceful best. Herd and keepers are traveling in the early morn. Awash in light, they appear through the mist, shadows long, breaths visible in the cold air, gait stiff, and uncertain from sleep. The woman is turned amiably toward her traveling companion. The dog jumping in the foreground is not troubling the cow nearby.

The pastoral idyll, originated in the third century by Theocritus in his accounts of the lives of Sicilian shepherds and painted so eloquently by Constable and Troyon, captured the imagination of an urban population longing for rural bliss. The perception and popularity of animals in parks and zoos, on the farm, as well as in paintings became the barometer of cultural change and the means to forge the broken bond with nature.

Late 18th- and early 19th-century poetry went down the same path. In “Home at Grasmere,” William Wordsworth spoke of a “mysterious” human-animal connection, “Mysteries of passion which have made, / And shall continue evermore to make…/ One brotherhood of all the human race.” And in “The Rime of the Ancient Mariner,” Samuel Taylor Coleridge recounted the travails of a hero, who having capriciously killed an albatross, suffered horribly and learned that, “He prayeth best, who loveth best / All things both great and small; / For the dear God who loveth us, / He made and loveth all” (*6*).

The uneasy balance between wilderness and civilization, upset in the 19th century by the loss of virgin land to cities, is still precarious, and our relationship with nature has grown more complex. Humans, animals, and goods have reached the remotest niches, eliminating the pastoral in ways Theocritus could not have imagined. And the mysterious “brotherhood” of all creatures envisioned by Wordsworth manifests itself in ubiquitous zoonotic connections.

Recent findings suggest that animals may be susceptible to human norovirus (*7*). In an unexpected turn on the way to market, bovine imports from the United Kingdom, a major source of human exposure to bovine spongiform encephalopathy, may have contributed to global risk for this disease (*8*). And genetic resistance of sheep with ARR/ARR prion genotype to the “classic scrapie” agent is not absolute, as we thought, despite culling and massive breeding efforts (*9*). As ever connected to and vulnerable in nature, we at times damage our fellow creatures to survive. Or, as Coleridge put it, “Ours is the reptile’s lot, much toil, much blame, / Manifold motions making little speed, / And to deform and kill the things whereon we feed” (*6*).
